# The Institutional Library

**Published:** 1914-11-07

**Authors:** 


### THE INSTITUTIONAL LIBRARY.

### "Heretical" Lectures.

The Operative Treatment of Fractures. By Sir W. Arbuthnot Lane, Bart., M.S., F.R.C.S. Second Edition. (London: The Medical Publishing Company, Ltd. 1914. Pp. 184+iv. Price 10s.)

Sir Arbuthnot Lane's views on the treatment of fractures come so sharply into conflict with what may be named the classical methods, that it is a great advantage to have them presented in a form accessible to the whole profession. Successive generations of students who have had the advantage of following his teaching at the bedside naturally become ready disciples. But outside this comparatively limited area there are large opportunities for crusading energy, and hence the present volume is to be heartily welcomed. For, let there be no mistake about it, the issue is a highly practical one, and of the first importance. There is no concealment of the challenge in such a statement as "I have described these badly united fractures of the femur to show how useless and inefficient are the usual methods of treatment of these injuries even in the hands of the most skilled and competent surgeons." Equally significant is the conclusion, based on a comparison of fractures found in subjects in the dissecting room with the teaching of recognised text-books: "Though experience had taught me to regard the statements in anatomical and surgical works with very strong suspicion, I was not prepared to find that the teaching of the causation, pathology, and the treatment of fractures and the results of such treatment was often absolutely false in almost every detail. It was evident that the displaced fragments of a broken bone were never, or hardly ever, restored to their normal position, and that the so-called 'setting of fractures' was a myth." It is not surprising that these conclusions when they were first expressed raised a storm of opposition, and we fancy that there are regions not a few in which the storm still continues. But "storms," from whatever quarter they blow, will not settle disputed issues. The final decision rests with practice and experience and facts, and in Sir Arbuthnot Lane's lectures all these are supplied in abundant measure. Whether they are conclusive is not for us to decide. But they certainly cannot be ignored by any practitioner who undertakes the charge of patients with broken bones. To all such there can be no difficulty in saying that it is a duty to study Sir Arbuthnot Lane's teaching as this is set forth, with a wealth of illustrations and much vigour of expression, in the present volume. Apart from its strictly practical aspect, the book has much interest as a record of the processes of reasoning and observation by which the author was led to the conclusions which he now advocates. These include, amongst other examinations, studies of the influences exerted by occupation and habit on joints, and students of arthritic disease may be advised to acquaint themselves with views which they may possibly consider heretical. But, in medicine at all events, we owe much to the heretics, and we are sure Sir Arbuthnot Lane will not shrink from plain phrases. He himself never avoids the "spade" for the sake of the "agricultural instrument." Altogether his lectures make enjoyable reading, and if we cannot profess to be fully convinced this may be due to the hardness of our hearts. Such, at least, we are sure, is the explanation which Sir Arbuthnot Lane will propose.

### A Medical Novel.

One Man's Way. A Novel. By Evelyn Dickinson. (London: George Allen and Co., Limited. 1914. Price 6s.)

Both as a romance of more than average value for holiday reading and as a novel with a distinct medical interest this book may claim a short notice in the technical press. The story itself is well told and is not encumbered with semi-philosophic dissertations, but goes on with a directness which is enhanced by the character of the incidents in the stormy career of the medical hero. This chief person of the drama is a country practitioner in a quiet retreat in Devonshire; the invasion of an evil female causes disaster, but ultimately the doctor rises to great heights as an alienist in Queen Anne Street, W., and continues to meet with a few more passionate adventures before the book closes in somewhat orthodox fashion. The author has succeeded in telling a good story in a style quite comfortable to read, and there are enough exciting episodes to please the most exacting reader.

### A Companion for Ward Work.

Elements of Surgical Diagnosis. By Sir Alfred Pearce Gould, K.C.V.O., M.S., F.R.C.S., and Eric Pearce Gould, M.Ch., F.R.C.S. Fourth Edition. (London: Cassell and Co., Ltd. 1914. Pp. 723+xiv. Price 10s. 6d. net.)

The first edition of this book was isued in 1884; it has been many times reprinted, and it now appears in a revised and enlarged fourth edition. Such a record of success seems to render superfluous the intervention of the reviewer, for the verdict of words must be weak when compared with the testimony of sales. Yet there would seem to be some want of courtesy in a mere formal announcement, and this more particularly to a trusted and well-known friend. For, indeed, like many of our readers, we can recall the day when, the terror of examination being in our soul, Pearce Gould's "Surgical Diagnosis" was a very present help in time of trouble. Not that the book belongs to a class whose object is to produce a show of knowledge without its substance. Very far from it, indeed. Rather it is a companion for that diligent and earnest ward work which is the only sure preparation for soundness of method, just as soundness of method is the avenue to accurate diagnosis. All this and many other helpful maxims are urged in the opening chapters, where the students of to-day, as of yesterday, may imbibe those useful principles without which surgical practice is a theory of chance and of shallowness. The application of these principles in the body of the work involves an enormous mass of detail, and this is handled with skill and judgment, and is generally well adapted to the students' needs. On individual points there may be some room for difference of opinion. Thus, while unilateral exaggeration of the knee-jerk is quoted as an aid in the localisation of an intracranial hæmorrhage, no mention is made of the significance of a unilateral Babinski's sign; a sixth-nerve paralysis is described as the result of certain cerebral tumours, without any allusion to its feeble localising value; and the effect on the visual field of tumours involving the several parts of the visual tract is wrongly described. The visual result of a tumour exerting pressure on the optic chiasma is, as everyone knows, blindness in the temporal half of each visual field, and not, as stated on p. 375, "blindness in the temporal part of each retina"; a similar error occurs in the description of the effect of a lesion involving the visual tract in its course through the internal capsule. We may, perhaps, venture to question the phrase "blindness of the retina." The retina, or any part of it, may, of course, be described as ineffective in function, or, perhaps, as paralysed; but blindness is a quality, not of the retina, but of the visual field; and we think it is a failure to appreciate this difference that has led to the confusion of statement to which we now direct attention. In any event such blunders are very bewildering for the unfortunate student. These criticisms, it is true, concern subjects somewhat outside the main surgical stream, but they certainly suggest the need for a more careful revision in such directions. So far as its chief purpose is concerned the value of the book is attested beyond challenge.

### The Sanatorium as a School.

THE TUBERCULOSIS HANDBOOK. By A. H. G. BURTON, M.D. (Lond.), D.P.H. (The Scientific Press, Limited. Price 2s. 6d. net.)

THIS little handbook is intended for the use of "health visitors and nurses, all social workers, heads of families, and others." It sets forth very simply such of the principal facts relating to tuberculosis as should be known by all engaged in public health work. The methods of infection are explained, and the recent report of the Royal College of Physicians on the infectivity of the disease is quoted in full. The danger arising from the cough of a consumptive patient is pointed out, but it is a matter of regret that the author does not emphasise the extreme importance of teaching patients invariably to hold paper handkerchiefs over the mouth during the act, whether expectoration is anticipated or not. Yet experience shows the great difficulty there is in getting patients to carry out this simple but all-important precaution. Treatment is dealt with under three heads: (1) Tuberculin; (2) Sanatorium; (3) Pneumo-thorax and other modes of treatment. These are simply and lucidly explained, but some may cavil at the precedence accorded to tuberculin. This is doubtless explained by the fact that the author is connected with dispensary work in which the results of tuberculin therapy appear to be rather more favourable than those obtained in residential institutions.

The satisfactory results that may be expected from a previous stay in a sanatorium, with all its educational advantages, are pointed out. In this connection it is difficult to understand why Dr. F. J. Smith, at the recent annual meeting of the British Medical Association, should deprecate the instruction given to sanatorium patients to take their own temperatures on leaving. The sanatorium is the proper place to teach patients to read a thermometer, and to emphasise the importance of the temperature in regulating rest and exercise. Without a temperature chart domiciliary and dispensary treatment cannot be scientifically carried out. In addition to pulmonary tuberculosis, the other forms of the disease with their appropriate treatment are briefly indicated, and a chapter is devoted to a description of the work of the tuberculosis dispensary and a nurse's duties in connection therewith. Recent legislation dealing with tuberculosis and the meaning of the Sanatorium Benefit clauses of the National Insurance Act are briefly explained. This little volume will be found extremely useful to all, whether intending to enter tuberculosis service or not, and should be read by all women engaged in district nursing, whose many duties will include attendance on patients receiving domiciliary treatment.

### A Guide to Medical Electricity.

PRACTICAL MEDICAL ELECTRICITY. By ALFRED C. NORMAN, M.D. (London: The Scientific Press, Limited. 1914. Pp. 226 + viii. Price 5s. net.)

THE ambition of Dr. Norman's book is to act as a link between works on medical electricity in the ordinary sense of this term and books written for the technical student. It keeps clearly in view the needs of the practitioner who desires to utilise the diagnostic and therapeutic values of electricity, while recognising that these values will not be obtained unless the scientific doctrines on which they are based are fully understood. We may say at once that, in our opinion, this programme has been very largely realised. Special pains have evidently been taken to obtain lucidity, for which the author deserves an ungrudging word of gratitude and recognition. Various forms of apparatus are illustrated, and diagrams are introduced to aid the descriptions given in the text. Of the applications of electricity for medical purposes, special attention is given to the production and uses of the *x*-rays. This section throughout has the authority of one who speaks with first-hand knowledge, and who has personally met—and overcome—the difficulties of the method. Indeed, we know of no book which meets so satisfactorily the wants of a practitioner who desires to equip himself in this branch of practice. It will be a surprise to some to read that good work may be done here with comparatively simple apparatus, provided only the user thoroughly understands his tools. The catalogues of the instrument makers seem to contradict this, but personally we place more confidence in Dr. Norman's experience. His book is a very helpful one, and ought to have a large success. It will assuredly be welcomed by the thousands of our readers who read the chapters as they first appeared in the pages of THE HOSPITAL.

PASTEUR AND AFTER PASTEUR. By STEPHEN PAGET, F.R.C.S. (Adam and Charles Black. Medical History Manual Series. Price 3s. 6d. net.)

THE difficult art of omission is most skilfully practised in this well-written and compact volume, which sets a standard for the series which it begins that the publishers will not find easy to maintain. Subject and author, in the case of Pasteur and Mr. Stephen Paget, are, as friends and enemies alike will admit, suited perfectly to each other. The result is a lucid, succinct summary of Pasteur's work from his early mathematical studies with "the pupil-teacher's board and lodging and twelve pounds a year at the College of Besançon," by way of the Ecole Normale, to the study of crystalline forms, and then ferments. At last his researches into the diseases of wines, and of infectious diseases in animals, grew out of one another till, as by organic development, the germ-theory of disease, and modern pathology and bacteriology with their immense reactions not merely in medicine, but in civilisation, as in Panama, evolved the new science of preventive medicine in all its Protean forms. It is wonderful to have packed so much, so clearly, into 150 pages, and the reader will regret only that the series was not of larger scope. Many interesting volumes are to follow in it, and as the announced list is capital as far as it goes, may we suggest that at least three volumes should be added to it?—one on Jenner and eighteenth-century inoculation, one on Hahnemann, and one, not to be overlooked, on the growth of the open-air treatment as first taught by George Bodington and early pioneers. Were it not an obvious *cliché*, we should describe Mr. Paget's capital book as a scientific romance, but lest the enemies of the Research Defence Society should seize on the misinterpretation to which the phrase is liable, we will call it merely a good working example of what a manual of medical history should be.

